# Interfaces in Atomic Layer Deposited Films: Opportunities and Challenges

**DOI:** 10.1002/smsc.202300060

**Published:** 2023-09-24

**Authors:** Syed Jazib Abbas Zaidi, Jae Chan Park, Ji Won Han, Ji Hyeon Choi, Muhammad Aanish Ali, Muhammad Abdul Basit, Tae Joo Park

**Affiliations:** ^1^ Department of Materials Science and Chemical Engineering Hanyang University Ansan 15588 Republic of Korea; ^2^ Department of Materials Science and Engineering Institute of Space Technology Islamabad 44000 Pakistan

**Keywords:** atomic layer deposition, freestanding 2D layers, high-*k* layers, seeding layers, 2D electron gas

## Abstract

Atomic layer deposition (ALD) is an effective method for precise layer‐wise growth of thin‐film materials and has allowed for substantial progress in a variety of fields. Advances in the technique have instigated high‐level interpretations of the relationship between nanostructure architecture and performance. An inherent part in the ALD of films is the underlying interfaces between each material, which plays a significant role in advanced electronics. Considering the impact of sandwiched substructures, it is appropriate to highlight opportunities and challenges faced by applications that rely on these interfaces. This review encompasses the current prospects and obstacles to further performance improvements in ALD‐generated interfaces. 2D electron gas, high‐*k* materials, freestanding layered structures, lattice matching, and seed layers, as well as prospects for future research, are explored.

## Graphical abstract

Considering the rise of atomic layer deposition (ALD) and its applications in electronics, it is essential to highlight mechanisms and latest research of interface‐related phenomena occurring between multilayered ALD films. As the quality of interfaces mostly determines the properties and performance of nanomaterial systems, this review can help researchers understand and develop ALD materials with tailored properties specific to applications.

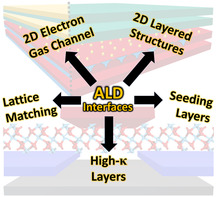

## 1 Introduction

The emergence of deposition techniques that can be controlled at the atomic level has accelerated progress in the semiconductor industry since the late 20th century. These bottom‐up techniques allow for the assembly of nanostructured thin films with desired properties for various applications.^[^ ^1^, ^2^ ^]^ However, an unintended consequence of layer‐by‐layer build‐up of these films is the formation of interfaces between each layer. Atomic layer deposition (ALD) is at the forefront of innovations in thin‐film technology, providing intricate control over deposited layers. In typical ALD, a substrate is placed in a vacuum reactor to which precursors are individually and alternatively introduced at certain temperatures and pressures with a purge cycle between each precursor exposure.^[^ ^3^ ^]^ A thorough understanding is required to select precursors, substrates, and temperature windows within which self‐saturating deposition occurs. A comprehensive account of the types of ALD and precursor chemistry, with a focus on metal sulfides and their applications, was explored previously.^[^ ^4^ ^]^ This review focuses on the interfacial interactions in thin films produced by ALD.

The term “interface” refers to the boundary between two phases—the separating boundary at which the previous layer ends and the next layer starts. Ideally, the two layers are chemically noninteractive and the interface acts as a sudden changeover to the next material. However, in practice, physical, chemical, and electronic interactions are inevitable in the contact region. These interactions cause various phenomena that suggest new avenues for research related to the interfaces. For example, the most apparent interactions may be those involving crystal lattices. Short et al.^[^ ^5^ ^]^ reported that their efforts to deposit multilayer films of ZnS and Cu_*x*_S revealed that the structure of films was determined by the whichever material was deposited first: Cu_2_S predominantly favors a monoclinic structure whereas CuS and ZnS prefer a hexagonal orientation.^[^ ^6^ ^]^ When ZnS was deposited first, a slight increase in CuS was seen due to the favored hexagonal structure, whereas when Cu_*x*_S was deposited first, a significantly lower amount of zinc was found compared to copper, likely as a result of delays in nucleation. This highlights the importance of interfaces, which can radically affect the second layer.

**Figure 1:**
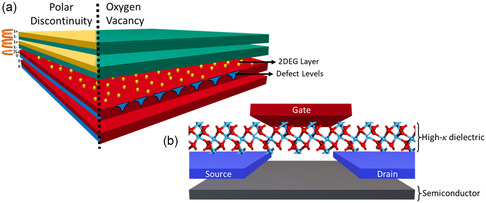
a,b) Schematic showing an exploded view of: a) 2DEG formation at the interface between two films and b) metal–oxide–semiconductor field‐effect transistor employing a high‐*κ* gate dielectric.

Interfaces also offer a wide array of opportunities for new research subjects, including the formation of 2D electron gas (2DEG) channels between two films and high‐*κ* materials in transistors and capacitors. A schematic of the internal structure of these devices is provided in **Figure** **1**. They will be discussed separately in later sections. These interfaces come with a variety of implications for the microelectronics industry.

ALD being a rapidly advancing technology with massive implications in electronics, it is crucial to understand the ALD interfaces generated in multilayered materials which can unlock the full potential of the technology. In addition, the properties and performance of many materials and devices are critically dependent on the quality of their interfaces. Specifically, multilayered materials can exhibit unique properties that are not present in their constituent materials, making the interface between them crucial for achieving desired properties. By understanding the importance of ALD interfaces in multilayered materials, researchers can optimize the interface and develop materials with tailored properties for a range of applications. This review will help consolidate the knowledge and identify gaps and prospects for its future. In general, interfacial interactions between multilayers can either provide beneficial properties that can be utilized in applications or present challenges that need to be overcome for optimal performance. This brief review discusses opportunities and challenges at the nanoscale, including 2DEG channels, high‐*κ* layers, freestanding layered structures, atomic lattice matching, and seeding layers.

## 2 Opportunities and Challenges

### 2.1 2DEG Channels

The term 2DEG refers to the conductive interface between insulating epitaxial layers, where electrons are constricted to that 2D plane. Ohtomo and Hwang^[^ ^7^ ^]^ initially reported finding the phenomenon between LaAlO_3_ and SrTiO_3_ in 2004. This sparked global research into the mechanisms behind it and subsequent applications such as field‐effect devices.^[^ ^8^ ^]^ The discovery was intriguing as the materials forming the interface are insulators. This seemingly anomalous mechanism behind interfacial 2DEG layers has been thoroughly researched in the scientific community. Two plausible mechanisms for the formation of 2DEG channels are avoiding polar catastrophe and generating oxygen vacancies. Both these mechanisms have been schematically shown in Figure 1a.

#### 2.1.1 Polar Catastrophe

The former maintains that, as the polar LaAlO_3_ layer abruptly meets the nonpolar SrTiO_3_ layer, an interfacial electronic reconstruction has to take place to avoid a polar catastrophe. If a supposedly unreconstructed electronic interface is analyzed, the electric potential should keep diverging as the thickness of the LaAlO_3_ layer increases. Upon reconstruction, the divergence disappears as a result of ½ e^−^ transfer to the interface, which produce the 2DEG. The latter mechanism involves uniformly distributed oxygen vacancies at the interface, which allows a shallow intragap donor level to form near the SrTiO_3_ conduction band. In either case, controlling a sharp interface is required to transform the interface from an insulator to a conductor. Inherently, combining a nonpolar layer with a polar layer will cause restructuring of the atoms leading to change in stoichiometry at the interface. However, Nakigawa et al.^[^ ^9^ ^]^ reported that chemical roughness can be exchanged for electronic roughness, and further fine‐tuning can be made by oxygen vacancies within the (001) LaAlO_3_/SrTiO_3_ interface.

#### 2.1.2 Oxygen Vacancies

Although it was once assumed that this mechanism was specific to the aforementioned materials, deposition processes such as ALD have allowed for the assembly of atomically precise interfaces, and researchers have exploited the latter mechanism to replicate 2DEG,^[^ ^10^, ^11^, ^12^, ^13^, ^14^, ^15^, ^16^ ^]^ even at binary metal–oxide interfaces, allowing for easier implementation in mass production. Seok et al.^[^ ^12^ ^]^ successfully realized the 2DEG phenomenon using ultrathin (<10 nm) binary oxide stacks of Al_2_O_3_ and TiO_2_. Prior studies depended on the use of single crystals or epitaxial layers, whereas Seok et al. utilized an ALD Al_2_O_3_/TiO_2_ heterostructure on an SiO_2_/Si substrate. As both layers consisted of nonpolar planes, the polar catastrophe mechanism does not play a role in this scenario. In addition, the study revealed the amorphous nature of ALD‐grown Al_2_O_3_, which eliminates the involvement of a lattice mismatch or polar discontinuity as an originating factor in the formation of 2DEG. The resulting 2DEG was restricted to a thickness of 2.2 nm and demonstrated an electrical performance equivalent to that of LaAlO_3_/SrTiO_3_ interfaces.

**Figure 2:**
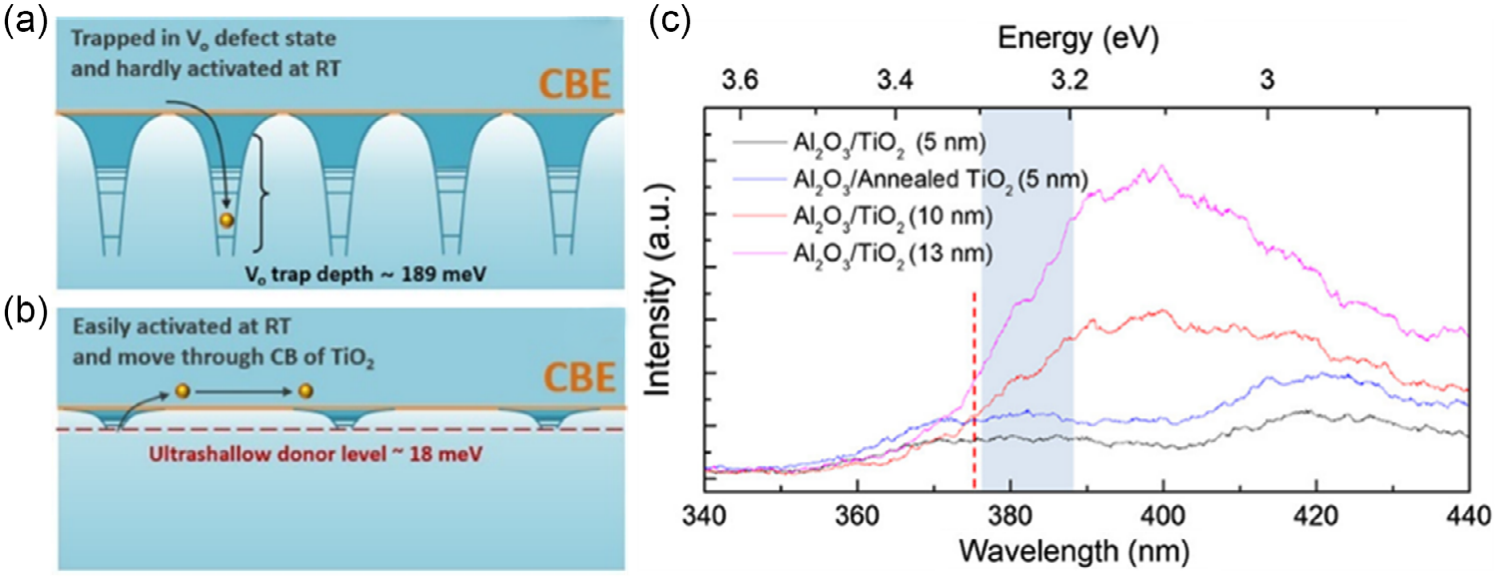
2DEG formation at the Al_2_O_3_/TiO_2_ interface. a) An amorphous TiO_2_ structure causes more donor levels at the interface, yet electrons are trapped at deep defect levels, b) crystalline bottom TiO_2_ leads to fewer but shallower donor levels closer to the conduction band edge, leading to the formation of 2DEG, and c) photoluminescence spectra at varying thicknesses and degrees of crystallinity of the bottom TiO_2_ layer. a–c) Reproduced with permission.^[^ ^11^ ^]^ Copyright 2020, The Authors, published by American Chemical Society.

The study confirmed the possibility of 2DEG using binary metal–oxide interfaces utilizing oxygen vacancies, but further investigation was needed in order to fully understand the mechanism that allowed formation of the 2DEG channel. In situ characterizations can allow observation during the experimental stage and provide better understanding. Hence, Seok et al. in a later study^[^ ^11^ ^]^ explored the creation of a 2DEG at the Al_2_O_3_/TiO_2_ interface using in situ techniques. The study found a significant dependence of the activation energy on electron release with the crystallinity of the bottom TiO_2_ layer. However, increased crystallinity had a detrimental effect on the density of oxygen vacancies. An amorphous TiO_2_ bottom layer had an activation energy of 189 meV, whereas that of an annealed polycrystalline layer was reduced to 18 meV, as shown in **Figure** **2**a,b. This 90% reduction in activation energy essentially allowed an extremely shallow donor state to readily provide electrons at room temperature. Seok et al. analyzed these donor levels through photoluminescence spectroscopy. The red line in Figure 2c indicates the wavelength at which band transition occurred. However, the study postulated that electron excitation via the defect level is more probable. In addition, shallower defect levels of self‐trapped emissions caused photoluminescence in a bandgap range of 3.2–3.3 eV. The possibility of 2DEG formation^[^ ^11^ ^]^ at a lower temperature of 300 °C, rather than the 750 °C needed for LaAlO_3_ growth,^[^ ^17^ ^]^ has led to widespread use in applications such as gas sensors.

**Figure 3:**
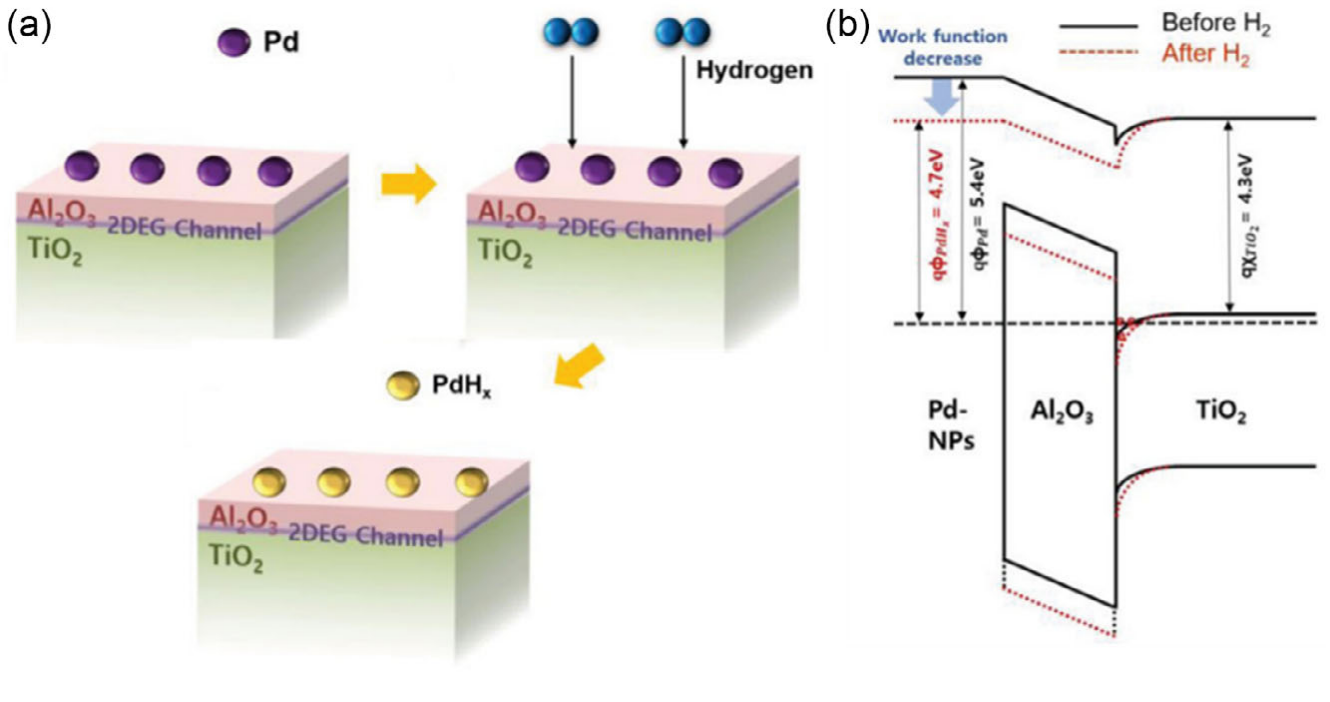
Palladium nanoparticles on Al_2_O_3_/TiO_2_. a) A mechanism for hydrogen gas detection and b) energy band changes upon hydrogen exposure. a,b) Reproduced with permission.^[^ ^15^ ^]^ Copyright 2018, Wiley‐VCH.

The formation of 2DEG channels via ALD is a relatively unfamiliar field and is constantly under development that is why applications for these devices are few. Potential applications involve gas sensors, biosensors, photodetectors, etc. Among these applications, Kim et al.^[^ ^15^ ^]^ used this oxide interface to create a transparent thin‐film hydrogen gas sensor using palladium nanoparticles on the Al_2_O_3_/TiO_2_ surface. The resulting device achieved high performance with a response time of less than 30 s. The mechanism responsible for H_2_ gas sensing was triggered by the formation of PdH_*x*_ on the surface, as illustrated in the schematic from Kim et al. (**Figure** **3**a), which caused a change in the work function from 5.4 to 4.7 eV. This in turn changed the conductance of the underlying 2DEG channel. The difference in the band diagram before (black) and after (red) exposure to H_2_ is shown in Figure 3b. The formation of PdH_*x*_ led to an increase in band bending, causing a transfer of electrons through the thin Al_2_O_3_ film to the Al_2_O_3_/TiO_2_ interface, decreasing the resistance in the 2DEG channel. This proposed mechanism was confirmed when Al_2_O_3_ film thickness increased, instigating a decrease in sensitivity.

#### 2.1.3 Piezoelectric Polarization

Interestingly, apart from the two common mechanisms discussed above, there might be other ways to induce disturbance at the interface that can lead to 2DEG. A recent study took advantage of the piezoelectric effect to form 2DEG at semiconductor interfaces. Jang et al.^[^ ^18^ ^]^ explored the possibility of a 2DEG channel between ALD‐BeO and ZnO via the polarization effect. The charge‐carrier density and mobility were higher than that of a previously studied Al_2_O_3_/TiO_2_ interface. In addition, the BeO/ZnO heterostructure showed a constant charge density of between 350 and 150 K, indicating weak temperature dependency. The study exploited the growth behavior of BeO on ZnO, which has a tendency to retain compressive strain,^[^ ^19^ ^]^ leading to piezoelectric polarization. The study theorizes that the resulting 2DEG is a consequence of spontaneous as well as piezoelectric polarization. Oxygen vacancies as a result of ALD may also be a factor. Further research into the mechanism is needed to fully understand the cause of 2DEG but the study provides an interesting idea for future investigations.

Our literature review has found that studies of 2DEG layers produced by ALD are relatively rare, and more investigation is necessary to understand such interfaces. Additional research should adopt first‐principle approaches that can provide superior insight into the interfaces and the 2DEG layer confined between them. A recent computational study by Park et al.^[^ ^16^ ^]^ showcased the mechanism of formation of oxygen vacancies when a trimethylaluminum, Al(CH_3_)_3_, precursor was exposed on the TiO_2_ surface leading to a 2DEG layer. A comprehensive study to identify viable candidates may be particularly valuable for future research.

### 2.2 High‐*κ* Layers

A consequence of scaling down transistors to keep up with Moore's law is a reduction in gate dielectric thickness for low‐energy, high‐performing complementary metal–oxide–semiconductor devices. However, conventional gate dielectrics such as SiO_2_ have reached their thickness limit as a result of gate leakage current. Alternates have been explored for decades,^[^ ^20^ ^]^ with HfO_2_ and ZrO_2_ the most notable examples.^[^ ^21^ ^]^ Replacing silicon with high‐electron‐mobility semiconductors has also attracted interest, with a higher success rate seen when switching to superior semiconductors (e.g., amorphous InGaZnO in the display industry^[^ ^22^ ^]^ and III–V semiconductors in integrated circuits^[^ ^23^ ^]^). An example of the application of these high‐*κ* layer is shown in Figure 1b where it is employed in a top‐gated metal–oxide–semiconductor field‐effect transistor.

#### 2.2.1 Fermi‐Level pinning

A preliminary problem known as Fermi‐level pinning occurs when two different materials with disparities in crystalline structure come into contact, leading to the formation of interfacial states. Metal–semiconductor interfaces are prone to Fermi‐level pinning. Especially among III–V semiconductors, the presence of interfacial defects such as elemental As, As dimers, and As and Ga dangling bonds leads to interfacial traps in the bandgap causing band‐bending and Fermi‐level pinning. Lower bandgap semiconductors such as InAs are less susceptible to these defects compared to high bandgap ones like GaAs. Although a smooth chemically noninteractive interface would be ideal, it is difficult to achieve. Hence, opting for a stable passivation layer has been considered a solution to the Fermi‐level issue.^[^ ^23^ ^]^ For GaAs surfaces, gaseous or liquid treatment with (NH_4_)_2_S has shown promising results. This allows for the formation of chemically stable –S‐terminated surfaces with minimal defects.^[^ ^24^, ^25^, ^26^, ^27^, ^28^, ^29^, ^30^, ^31^, ^32^ ^]^ However, other III–V surfaces are not easily passivated where (NH_4_)_2_S treatment leads to the formation of surface oxides.^[^ ^33^ ^]^ In addition, other interfacial passivation layers, such as germanium,^[^ ^34^ ^]^ silicon, and Si/Si_3_N_4_,^[^ ^35^ ^]^ tend to exhibit superior interfacial properties on GaAs and InGaAs.

#### 2.2.2 Interface Clean‐Up

A subsequent problem is the need for clean‐up of native surface oxides in order to ensure the sudden changeover from semiconductor to high‐*κ* layer is possible. However, an interesting phenomenon occurs when the metal precursor is exposed to the semiconductor surface known as self‐cleaning. The precursor molecules such alkylamides react with surface oxides and form volatile byproducts that are purged out. Later, the surface can be saturated with the precursor molecules for typical ALD growth of the high‐*κ* layer. Klejna et al.^[^ ^36^ ^]^ described the phenomenon of thinning of native oxides, which was observed previously in ALD of Al_2_O_3_,^[^ ^37^ ^]^ HfO_2_,^[^ ^38^, ^39^ ^]^ TiO_2_,^[^ ^40^, ^41^ ^]^ and Ta_2_O_5_.^[^ ^42^ ^]^ The study subsequently conducted a detailed investigation using density functional theory (DFT) tools. The authors postulated that metal precursor ligands were attracted to the native oxides on the surface. Common metal precursor types such as alkylamides, methyls, and chlorides have since been compared to identify the most chemically viable precursor for clean‐up. Although methyl precursors achieved the best performance, the ALD precursor type remains unstable. Alkylamides have a similar self‐cleaning mechanism for scavenging oxygen from weaker native oxides, but the bulky nature of the molecules leads to steric hindrance, which limits the process.^[^ ^36^ ^]^ In a contrary finding, Li et al.^[^ ^43^ ^]^ highlighted the adverse effect of exposure to trimethyl‐Al precursor on InGaZnO during deposition of an AlO_*x*_ dielectric, which caused interfacial defects, clockwise hysteresis, and stress degradations. The study introduced N_2_O plasma as a countermeasure to improve performance and stability. Although these studies postulated the possibility of self‐cleaning process in ALD of high‐*κ* layers, a deeper understanding was needed to study the underlying phenomena. Hence, in situ characterizations techniques integrated with ALD were employed to offer definitive evidence and mechanism behind the self‐cleaning phenomenon.

**Figure 4:**
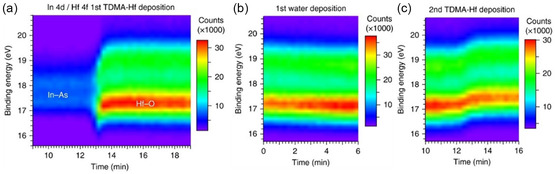
Chemical reactions at the InAs surface upon HfO_2_ ALD showing time‐dependent XPS evolution of the surface chemistry with exposure to TDMA‐Hf (a), H_2_O (b), and TDMA‐Hf once again (c). a–c) Reproduced under the terms of the CC‐BY Creative Commons Attribution 4.0 International license (https://creativecommons.org/licenses/by/4.0).^[^ ^44^ ^]^ Copyright 2018, The Authors, published by Springer Nature.

Timm et al.^[^ ^44^ ^]^ further explored practical self‐cleaning reactions in ALD of HfO_2_ reported that the driving force of the cleaning mechanism was molecular adsorption rather than the subsequent dielectric oxide formation postulated by Klejna et al.^[^ ^36^ ^]^ Surface changes were analyzed with the help of time‐resolved photoelectron spectroscopy data collected during the HfO_2_ ALD process. A more intense hafnium signal was detected in place of the In‐As signal upon exposure to a tetrakis(dimethylamino) (TDMA)‐Hf precursor (**Figure** **4**a). The hafnium peak shifted toward lower and higher binding energies when exposed to H_2_O and a second TDMA‐Hf step, respectively (Figure 4b,c). The researchers reported that, in the case of chemical reactions, the peaks for hafnium and oxygen typically shift independently of each other, indicating changes in oxidation states. However, both peaks shifted together during each step rather than in opposite directions (as in redox reactions), indicating an increase in hydroxide groups and reducing the ionic character of Hf—O bonds.

### 2.3 Freestanding 2D Layered Structures

One area of extensive research involving ALD is the development of freestanding 2D layered structures with unique properties and potential applications.^[^ ^45^ ^]^ Graphene has been a significant inspiration in the discovery of several other layered materials.^[^ ^46^ ^]^ Promising materials include MoS_2_,^[^ ^47^ ^]^ WS_2_,^[^ ^48^ ^]^ MoO_3_,^[^ ^49^ ^]^ and SnS_*x*_,^[^ ^50^ ^]^ which possess distinctive physical, chemical, and electronic features. These structures have strongly bonded in‐plane atoms constructing a 2D layer whereas each out‐of‐plane layer is joined with van der Waals forces. By definition, it seems obvious that ALD process which involves layer‐wise deposition should be a good match for the development of 2D materials. An added advantage of using ALD would be deposition at lower temperature rather than the high temperatures needed for chemical vapor deposition (CVD) processes. However, a drawback is the complex precursor chemistry and parameters involved in growing such structures.

Cai et al.^[^ ^51^ ^]^ classified three different approaches (R1, R2, and R3) to growing 2D materials using ALD and discussed their potential advantages. R1 is a traditional ALD method in which each successive layer is grown in a continuous fashion. In comparison, R2 is a temperature‐dependent, self‐limiting technique in which the deposition of layers ceases as soon as a desired temperature is reached, and ALD cycles therefore do not dictate the number of layers. R3 is a two‐stage process in which the film of a suitable precursor is first deposited using ALD at lower temperatures followed by high‐temperature treatment, resulting in the formation of 2D material. These methods provide a variety of benefits, such as precise control over the number of layers, increased crystallinity of 2D materials, and wafer‐scale uniformity.

#### 2.3.1 Out‐Of‐Plane Growth

Despite the various approaches, growth of 2D layered structures via ALD has been a challenge. A notable issue is the out‐of‐plane growth hindering deposition of layers as vertical growth of nanostructures dominates rather than formation of lateral layers. The reason being that general ALD process conditions are designed to promote chemical bonding rather than physical interactions like van der Waals needed for 2D layers growth. Hence, following these approaches, prevalent growth of vertical nanostructures is predictable. This not only hinders further growth but also conductivity of the materials. Considering that these structures have a major application in electronics, film conductivity is a major factor among these films. Previous research^[^ ^52^, ^53^ ^]^ has demonstrated that, by introducing a plasma step in conjunction with regular ALD, out‐of‐plane vertical nanostructures in 2D materials can be prevented or altered by chemically etching nucleating sites, increasing the material's grain size.

#### 2.3.2 Use of Inhibitors

**Figure 5:**
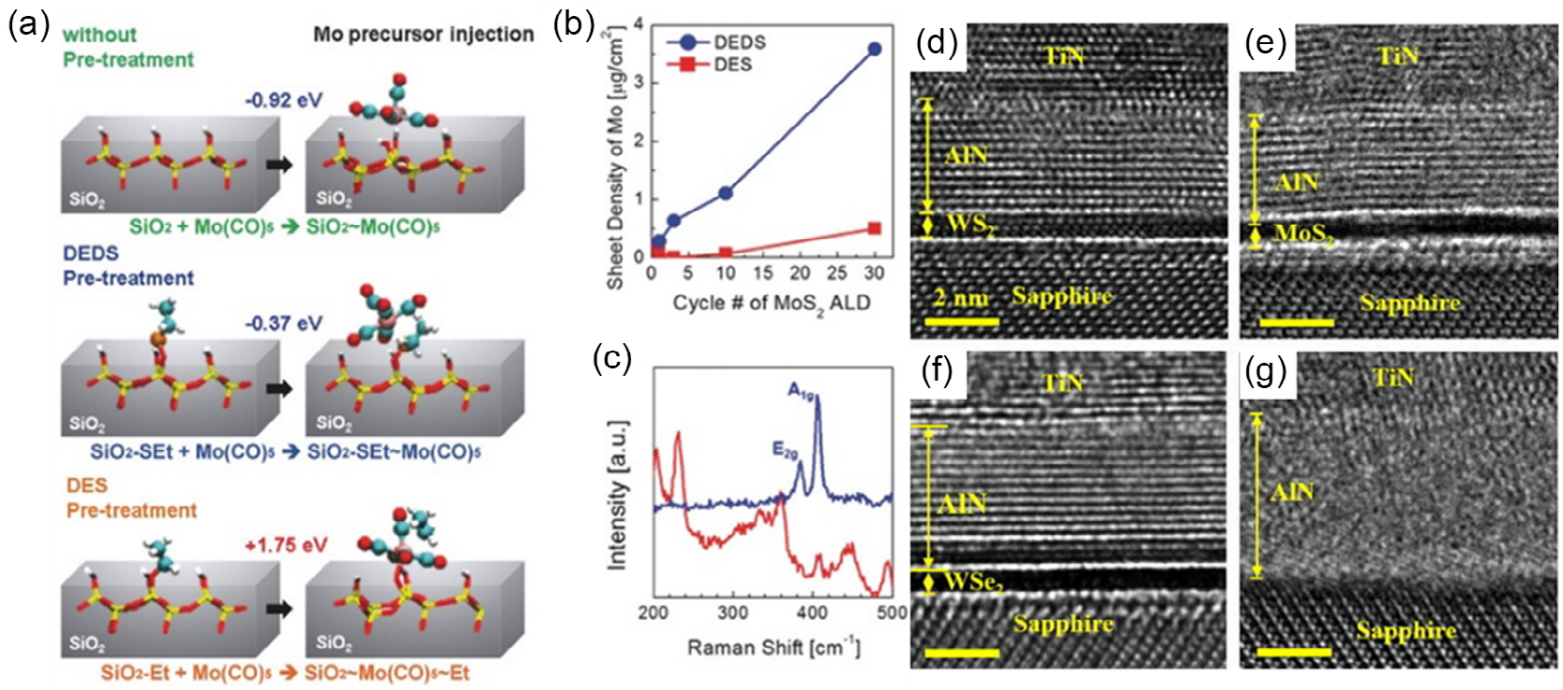
ALD of 2D freestanding materials. a) Molybdenum precursor adsorption and bond formation on various SiO_2_ substrate surfaces i.e., pristine, DEDS treated, and DES treated, b) a comparison of sheet density, and c) Raman spectra of MoS_2_ layers deposited via DEDS at 250 °C and DES at 350 °C. a–c) Reproduced with permission.^[^ ^54^ ^]^ Copyright 2017, Wiley‐VCH Verlag. d–g) HRTEM images depict TiN/AlN deposition in a single experiment on WS2 (d), MoS_2_ (e), WSe_2_ (f), and sapphire (g). d–g) Reproduced with permission.^[^ ^60^ ^]^ Copyright 2022, AIP Publishing.

As previously mentioned, freestanding 2D layered materials are connected via physical van der Waals forces rather than chemical bonds, hence preventing the formation of chemical bonds can help in the formation of van der Waals materials. Controlling nucleation by using inhibitors is a way to disallow the chemisorption of precursors and promote van der Waals structure formation. In an example of MoS_2_ films on SiO_2_, the Mo precursor exposed to the substrate is chemisorbed onto the surface forming stable Mo—O bonds. Inhibitors can passivate the surface disallowing the chemisorption and promoting 2D layered growth. Jeon et al.^[^ ^54^ ^]^ studied MoS_2_ at atomic‐scale thicknesses for high‐performance nanoelectronics, focusing on thermodynamics and kinetics of molybdenum precursor adsorption on a surface‐modified substrate using first‐principles calculations based on DFT. The study discusses two different pretreatments, diethyl sulfide (DES) as an inhibitor and diethyl disulfide (DEDS) as an activator, which are used to investigate the adsorption of Mo(CO)_6_ on SiO_2_ surfaces, as shown in **Figure** **5**a. In a conventional MoS_2_ ALD deposition process, Mo—O bonds are formed when the Mo(CO)_6_ precursor is exposed to the SiO_2_ without any pretreatment (Figure 5a top). When surface is pretreated with DEDS, the chemisorption of Mo(CO)_6_ precursor is also promoted by formation of Mo—S bond (Figure 5a middle). Whereas upon pretreatment with DES, a stable C—O bond is formed which makes it kinetically unstable for the chemisorption of Mo(CO)_6_ precursor (Figure 5a bottom). This suggests that DES pretreatment inhibits Mo(CO)_6_ by passivating the adsorption sites, while DEDS pretreatment facilitates Mo(CO)_6_ adsorption. It is important to note that the study recommends the use of DEDS as the counter reactant with Mo(CO)_6_ precursor in the conventional ALD process as it provides higher growth rate without any incubation period compared to DES as evident in Figure 5b. In addition, the Raman spectra in Figure 5c validate the formation of a layered structure, which is indicated by the presence of E_2g_ (in‐plane) and A_1g_ (out‐of‐plane) modes at 383 and 408 cm^−1^, respectively. Results of previous studies, such as those involved MoS_2_ films synthesized using dimethyl disulfide^[^ ^55^ ^]^ and H_2_S,^[^ ^56^, ^57^ ^]^ are in fair agreement with these results.

#### 2.3.3 Grain Size

Achieving a larger grain size has presented a challenge for 2D layered materials. Although using higher deposition temperatures in CVD has allowed overcoming the issue, the downside is the incompatibility of the methods with BEOL processes.^[^ ^58^ ^]^ ALD for 2D layered growth has shown promise in this regard but larger grain size remains an issue. An effective strategy for a larger grain size would be to lessen the nucleation density at the substrate surface. To address this, Yang and Liu^[^ ^59^ ^]^ introduced a new technique that employs nickel‐foam‐based trickle‐flow ALD to synthesize 2D MoS_2_ with a larger grain size. Their research indicates that, by using nickel foam 1 mm thick on top of the substrate with a 2 mm gap between the foam and the substrate and carrying out ALD at 460 °C, they were able to produce MoS_2_ with grain sizes of up to 420 nm (monolayer sample) and 400 nm (five‐layer sample). Their study also revealed that placing nickel foam of a particular thickness on top of the substrate creates a trickle‐fluidization source flow that lowers nucleation density. This results in the typical ALD mechanism of vertical growth on a large scale, leading to the expansion of MoS_2_ grain sizes due to steric hindrance following planar parallel growth around the crystal nucleus. The nickel foam also improved heat transfer around the substrate, leading to a stable temperature field and improving crystallinity. These findings suggest that trickle ALD is a promising technique for producing high‐quality, large‐scale 2D MoS_2_, which has important implications for future research into 2D materials.

#### 2.3.4 Substrate Preference

Taking advantage of the preference of substrate upon which the 2D layered structure is being deposited can be an interesting opportunity. Considering the structure of these films is joined via van der Waals interactions rather than chemical bonding, lattice matching conditions can be reduced if the substrate itself is a van der Waals material. Further review on lattice matching is provided in Section 2.4. Typical growth of ALD film consists of chemical bonding which subsequently leads to an interfacial layer acting as a buffer between the substrate and grown film. However, choosing a suitable substrate, i.e., van der Waals material, can promote easier deposition of 2D layered materials. Recently, Chang et al.^[^ ^60^ ^]^ reported the use of low‐temperature ALD to grow 2D hexagonal aluminum nitride (h‐AlN) on transition‐metal dichalcogenide (TMD) monolayers, which are effective semiconductors for high‐performance integrated circuitry. Van der Waals epitaxy was used to achieve epitaxial 2D layered h‐AlN. Figure 5d–g shows cross‐sectional high‐resolution transmission electronic microscope (HR‐TEM) micrographs of TiN/AlN films on various substrates. The deposition of TiN/AlN ALD films on WS_2_/sapphire, MoS_2_/sapphire, and WSe_2_/sapphire is illustrated in Figure 5d–f, while Figure 5g shows the amorphous growth of an AlN film on the sapphire substrate. The growth rate of the layered h‐AlN film on TMDs was slower than that on a sapphire substrate, likely due to the chemically inactive surface of TMDs, which only allows for van der Waals physisorption during layered h‐AlN deposition. TMDs were therefore responsible for the structural features of h‐AlN instead of the sapphire substrate. The results suggest that h‐AlN can serve as a suitable interfacial layer between TMD and high‐*k* 2D insulators.

#### 2.3.5 Comparison with CVD

**Figure 6:**
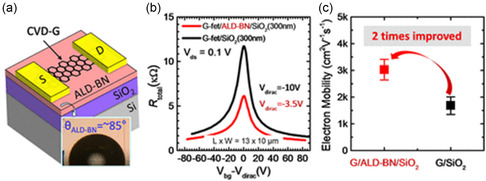
ALD of 2D BN for electronics. a) Graphene field‐effect transistor on BN, b) transfer curves, and c) electron mobility of the device on pristine SiO_2_ compared to an ALD BN substrate. a–c) Reproduced with permission.^[^ ^61^ ^]^ Copyright 2020, American Chemical Society.

Compared to the commonly utilized method for 2D layered growth, i.e., CVD, ALD offers less complexity, lower temperature, and precise control over thickness. Although CVD has allowed growth of large‐scale and quality 2D layered films, it requires a high energy budget and a complicated transfer process that is prone to defects and residues at the interface. A direct stacking method such as ALD solves these issues. A relevant study was conducted by Lee et al.,^[^ ^61^ ^]^ who reported employing ALD on BN at 600 °C using a substrate of SiO_2_. Precursors used were BCl_3_ and NH_3_. The deposited films were uniform, smooth, and showed ordered crystallinity at nanoscales. In contrast to hexagonal boron nitride (h‐BN) film grown by CVD, ALD‐BN resulted in considerably greater growth. A decrease in surface charge density coupled with increased inertness of ALD‐BN surface doubled the carrier mobility of graphene field‐effect transistors (G‐FETs). Moreover, similar dielectric properties were recorded for ALD‐BN and SiO_2_. **Figure** **6**a is a schematic of a typical G‐FET device. Graphene was transferred on SiO_2_ (thickness of 300 nm) containing an as‐deposited thin film of ALD‐BN. The aim was to investigate the insulating capability of layered BN deposited using ALD for application in 2D devices. The transfer curves of G‐FETs on two substrates, namely, ALD‐BN/SiO_2_ and bare SiO_2_, with 30 nm Al_2_O_3_ passivation, are shown in Figure 6b. The G‐FETs on bare SiO_2_ exhibited a Dirac point shift of −10 V, while the G‐FETs with the ALD‐BN film showed a shift of −3.5 V. The identical passivation in both devices emphasizes the significance of the high doping induced *V*_dirac_ shift in the graphene channel. The shift can be ascribed to the underlying dielectric, which suggests that ALD‐BN‐related defects, such as grain boundaries and surface oxidation, from the ex situ graphene transfer process caused a smaller amount of unintentional doping in the graphene. The mobility of the graphene device on ALD‐BN was considerably higher than that of the graphene device on bare SiO_2_ (Figure 6c). As a result of ALD‐BN, the lowered surface defects such as grain boundaries at the interface cause relatively smaller amount of unintentional doping of transferred graphene which lead to a better quality device. The improvement in mobility is also noteworthy, indicating that ALD‐BN can enhance the performance of graphene devices due to the inert surface of layered BN, which is less susceptible to surface scattering compared with SiO_2_. The process can be carried out at relatively low temperatures, making it convenient and feasible for industrial applications.

#### 2.3.6 Computational and In Situ Studies

Theoretically, ALD can form an ideal 2D layered film on top of the substrate with no defects but in practical applications deviations from the perfect interface are bound to occur. Hence, studying the interface through computational and experimental means is essential. In this regard, Lee et al.^[^ ^62^ ^]^ highlighted a concern with the synthesis of freestanding monolayers of ALD MoS_2_ on SiO_2_ due to S–O van der Waals interactions. Their study suggests a strong electronic interaction at the interface in contrast to the ideal 2D structure. Although SiO_2_ is an insulator, the oxygen ions at the interface contributed to valence as well as conduction bands, leading to electronic interactions. To further advance the understanding of ideal monolayers of MoS_2_, Kim et al.^[^ ^63^ ^]^ modified a typical process of ALD MoS_2_ using a Mo(CO)_6_ precursor so that an absolute MoS_2_ monolayer could be achieved in a single ALD cycle. This process hinged on two important conditions: over‐adsorption and absorbate control. The prior condition meant that there needed to be an excess of precursor exposure to avoid steric hindrance. This was achieved at a temperature slightly higher than that of a stable ALD window for partial decomposition of the precursor, ensuring over‐adsorption. The latter allowed for control over the adsorbate (precursor nuclei) forming on the substrate surface, which can correspond to one MoS_2_ monolayer. Splitting the feeding time into segments allowed for this control followed by annealing in H_2_S environment. This method led to the formation of a wafer‐scale 2D layered structure with a highly uniform crystalline film.

### 2.4 Lattice Matching

High‐quality crystalline films are crucial for electronic applications of thin films produced by ALD. Accumulation of strain energy can be a challenging issue during the deposition of thin films due to several factors, such as lattice mismatch, incomplete reactions, and impurities. When the crystal lattice of a substrate differs from that of a deposited thin film, a phenomenon known as lattice mismatch occurs, which can result in the accumulation of strain energy.^[^ ^64^ ^]^ Irreversible deformation and defect generation can occur due to lattice mismatch, posing a significant obstacle to the manufacturing of high‐quality thin films for electronic applications. Moreover, incomplete reactions during the ALD process can contribute to nonuniformities and defects in the film, leading to a decline in the crystallinity of the film.^[^ ^44^ ^]^ Optimizing the process conditions, including temperature, precursor pulse duration, and exposure time, can enhance the reaction kinetics and promote complete surface saturation, addressing this challenge. In addition, impurities in the deposition environment can introduce defects that alter the electronic properties of the film, even in trace amounts, affecting its crystallinity.^[^ ^65^ ^]^ It is therefore essential to use high‐purity precursors and maintain a clean deposition environment to produce high‐quality crystalline films. To achieve high‐quality films for electronic applications, it is necessary to overcome challenges in the ALD process such as lattice mismatch, incomplete reactions, and impurities, as the crystal structure of the substrate can significantly affect the nucleation and growth of the ALD film.

The degree of lattice matching between the substrate and the film plays a crucial role in determining the crystallinity of the film. A substrate with a crystal structure similar to that of the ALD film will result in superior crystallinity due to favorable lattice matching and reduced strain at the interface. An example of this was provided in the previous Section 2.3. In contrast, a substrate with a different crystal structure can either result in a lower degree of crystallinity due to the formation of defects and mismatched interfaces^[^ ^66^ ^]^ or trigger the formation of a different crystalline structure of the deposited film.^[^ ^67^, ^68^ ^]^

#### 2.4.1 Compatible Crystal Structures

Choosing a substrate with a crystal structure suitable for the intended ALD film can lead to improved film quality and device performance. Considering the large difference in dielectric constant between rutile and anatase‐phase TiO_2_, postdeposition annealing is usually required to convert the anatase phase that is stable at room temperature to a high‐*κ* rutile phase.^[^ ^69^ ^]^ However, Kim et al.^[^ ^67^ ^]^ discovered that ALD TiO_2_ films grown on Ru/RuO_2_ electrodes formed rutile structures without a high‐temperature annealing step, emphasizing the influence of the substrate crystal structure on the deposited film. The reason for the growth of rutile structure of TiO_2_ can be attributed to crystallographic similarity between the substrate surface, i.e., RuO_2_ and the rutile TiO_2_, which leads to desirable crystal structure growth. A similar concept was applied by Lee et al.^[^ ^68^ ^]^ recently while introducing MoO_2_ as a new oxide electrode for dynamic random‐access memory (DRAM) capacitors. The main benefit of using MoO_2_, apart from inducing the development of rutile TiO_2_, is its thermal stability compared with RuO_2_ and SrRuO_2_. This is significant because DRAM capacitors suffer from thermal stresses. The resulting rutile film exhibited outstanding coherency without the formation of intermediates or oxidation of MoO_2_ to MoO_3_. Similarly, among multilayered film ALD, the growth of the secondary film is dependent on the structural properties of the preceding film. This can be utilized to achieve specific orientational growth for desired properties given that the films are of compatible materials. Short et al.^[^ ^5^ ^]^ used ALD to assemble multilayer film stacks of ZnS and Cu_*x*_S to create a nanoscale copper–zinc–sulfide composite, investigate the impact of initial layer growth on a Cu_*x*_S/ZnS nanolaminate structure, and assess whether thicker layers could improve the stability of film growth. The thickness of the layers ranged from 2 to 20 nm, and film structure was found to be determined by the first material deposited in thicker films, whereas the thinnest layers were dominated by Cu_*x*_S. The thickness requirement might be needed to overcome the interfacial layer formed between the substrate and the first layer deposited. Hence, the phenomenon was not observable in thinner films.

#### 2.4.2 Mismatched Crystal Structures

**Figure 7:**
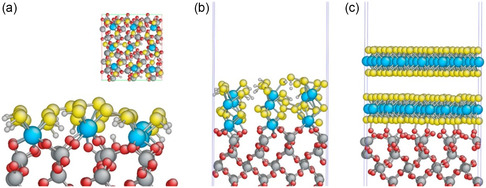
Density function theory applied to the formation of freestanding MoS_2_ layers on an SiO_2_ substrate. a) A buffer layer at the surface during an initial ALD cycle (inset shows top view), b) a subsequent layer of MoS_2_ leading to amorphous deposition on the buffer layer, and c) ideal deposition of a MoS_2_ freestanding layer at the interface. a–c) Reproduced with permission.^[^ ^70^ ^]^ Copyright 2018, AIP Publishing.

In contrast, when the substrate and grown film have unmatching crystal structures, it is likely that a buffer layer is formed at the interface as a transition. This has been observed during the ALD process that the first few cycles will deposit an amorphous layer to overcome the mismatch between the two materials. Shirazi et al.^[^ ^70^ ^]^ employed DFT calculations to investigate the formation of a buffer layer during the initial stages of ALD of MoS_2_ on a SiO_2_ (001) surface and proposed alternative ALD chemistries that facilitate the formation of a freestanding 2D‐MoS_2_ nanolayer with preserved optoelectrical properties. This was achieved by assembling an underpinned building block with molybdenum atoms underpinned by sulfur atoms, preventing the formation of a buffer layer. **Figure** **7**a depicts the mismatch between the number of molybdenum atoms in the SiO_2_ surface area after traditional ALD reactions and the number of molybdenum atoms that should be located in the same area. Chemical reactions of Mo(NMe_2_)_2_(NtBu)_2_/H_2_S on SiO_2_(001) resulted in the formation of an amorphous MoS_2_ buffer layer on the SiO_2_ surface instead of freestanding MoS_2_ layers because the structure of the formed MoS_2_ did not match the crystalline SiO_2_ surface after the initial cycles of ALD. The optimized formation of nine molybdenum atoms occurred at the interface during the ALD process; ideally, 28 molybdenum atoms should be situated in the same area. Figure 7b depicts the morphology of the amorphous thin film at the interface, with only two layers considered. This amorphous structure can spread along the *z*‐direction through different numbers of MoS_2_ layers. Figure 7c depicts the transformation of the amorphous thin film into a few freestanding layers of crystalline MoS_2_ at the SiO_2_ interface after annealing in a sulfur‐containing atmosphere. For direct fabrication of electronic devices, the deposited thin film should be as thin as a monolayer of MoS_2_ and should readily obtain the structure of the freestanding layered MoS_2_ at a low temperature. These studies highlight the significance of the first few layers during ALD. These interfacial layers can influence the physical, chemical, and electronic properties of the film.

#### 2.4.3 In Situ Investigations

**Figure 8:**
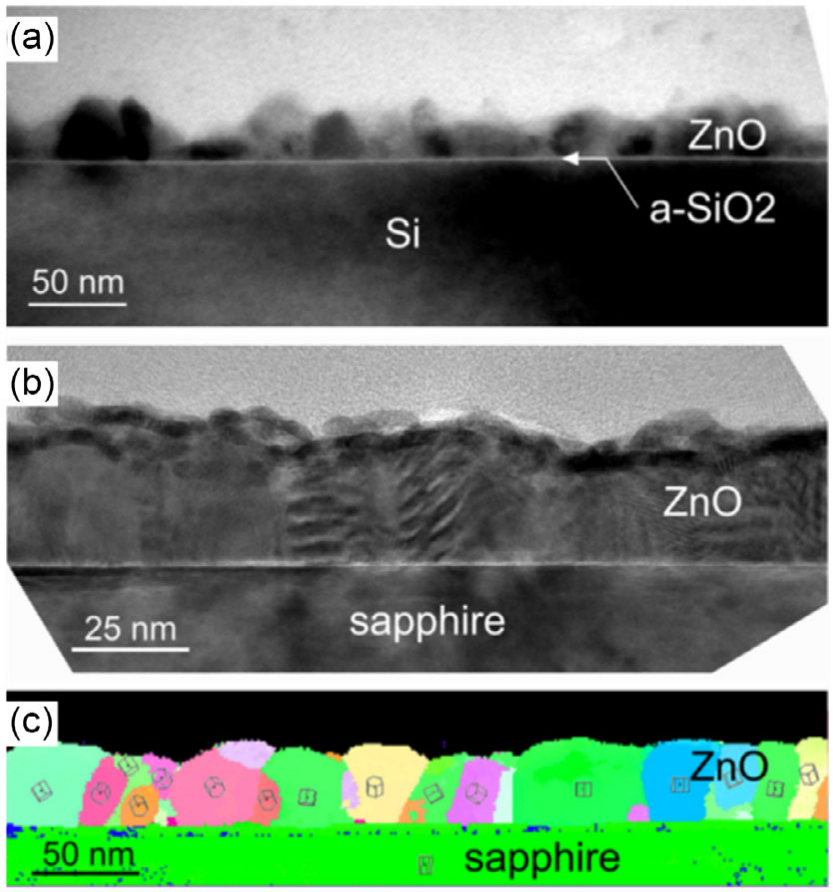
Development of crystalline structures of ZnO after 200 ALD cycles. a,b) Cross‐sectional TEM images showcased for deposition on a‐SiO_2_ (a) and c‐Al_2_O_3_ (b) substrates. c) A crystal orientation map for ZnO/Al_2_O_3_. a–c) Reproduced with permission.^[^ ^71^ ^]^ Copyright 2020, American Chemical Society.

Apart from computational studies, in situ studies during ALD can also help in gaining information about the initial layers that form the interface. ALD growth comprises of nucleation and growth similar to other processes. Boichot et al.^[^ ^71^ ^]^ examined the initial 10 cycles of ZnO thin‐film growth using ALD. They used in situ synchrotron X‐ray techniques to analyze the chemical and structural changes during the growth process. The study confirmed that ZnO grown on a‐SiO_2_ and c‐Al_2_O_3_ substrates shows nucleation during the first cycle and coalescence during the second cycle and nucleation density heavily relies on the hydroxyl coverage as well as the longer pulse times. In addition, the structure created during the coalescence phase has a significant impact on the overall microstructure of the film. The crystalline texture of the films was also found to be heavily influenced by interfacial bonds. The study suggests that the nucleation and growth of nanoscale islands during the initial stages of ALD film formation are critical in determining the resulting film's properties. **Figure** **8**a,b depicts a cross‐sectional TEM micrograph of ZnO layers after 200 growth cycles for ZnO/a‐SiO_2_ and ZnO/c‐Al_2_O_3_, respectively. In the case of ZnO on a‐SiO_2_, the image confirms the existence of a continuous amorphous silica layer approximately 1 nm thick on the surface of the substrate. The film comprises rough surfaces and grains ranging in size from 15 nm to 25 nm. The ZnO layer deposited on c‐Al_2_O_3_ had grains of similar sizes (approximately 15–20 nm), but with a smoother surface. Applying orientation mapping (Figure 8c) to approximately 50 ZnO crystallites revealed that most grains had an epitaxial relationship with the c‐Al_2_O_3_ substrate. The images also confirmed columnar microstructures for both ZnO films. Additionally, the research found differences in crystalline texture when growing ZnO on silicon with a native oxide versus growing it on Al_2_O_3_(001), indicating variations in interfacial bonds. The strong Al—O bonds upon exposure to the Zn precursor help lower the interfacial energy which relates to the (001) alignment domains. These findings offer valuable insights into the ALD process, which can be elaborated using computational methods and help in high‐permittivity dielectrics for semiconductors and solid‐oxide fuel cells.

**Figure 9:**
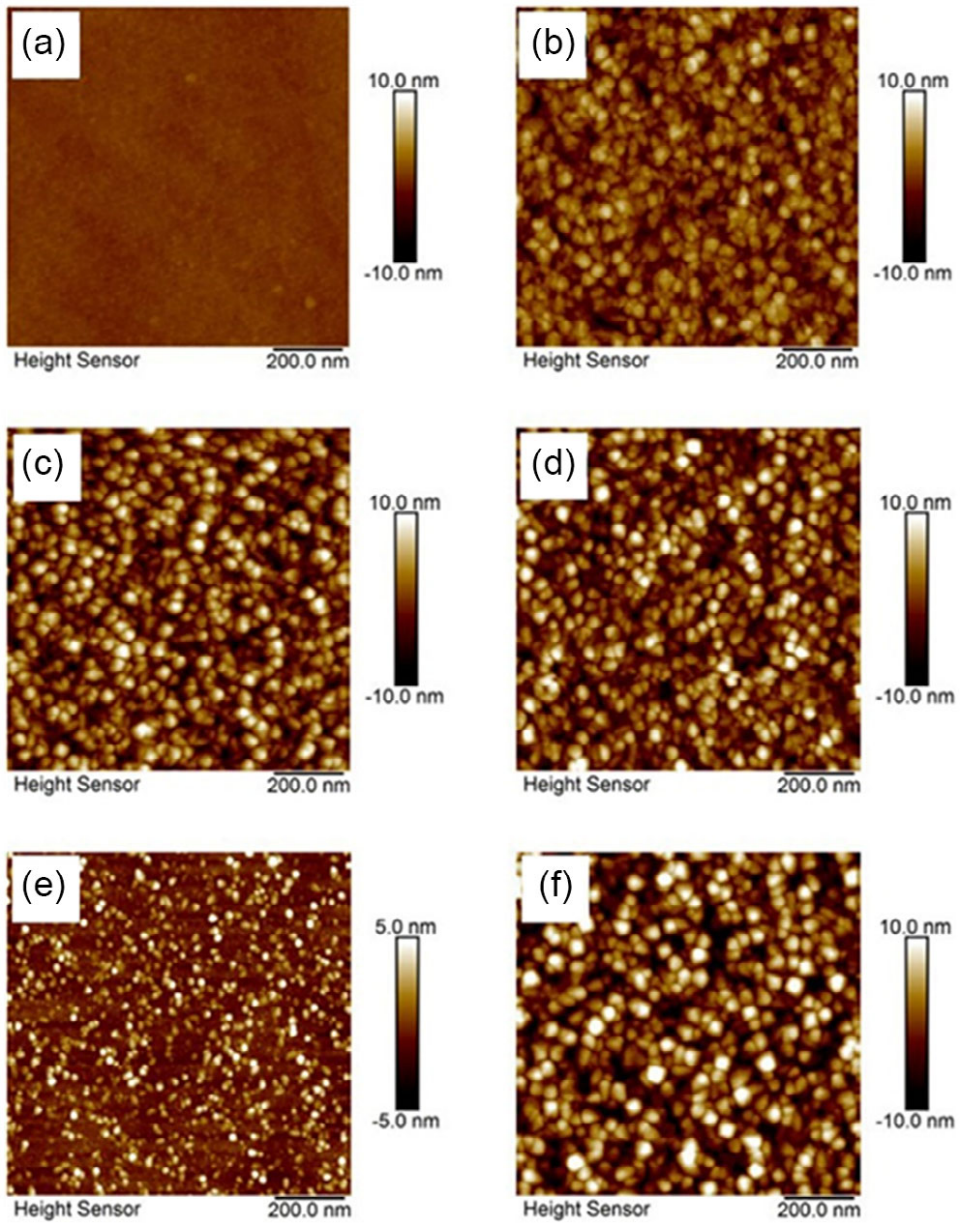
a–f) AFM images showing ALD ZrO_2_ films deposited at 150 °C (a) and 350 °C (b); deposited at 250 °C followed by annealing at 400 °C (c) and 1000 °C (d); and deposited at 250 °C with 200 cycles (e) and 800 cycles (f). Reproduced under the terms of the CC‐BY Creative Commons Attribution 4.0 International license (https://www.creativecommons.org/licenses/by/4.0).^[^ ^72^ ^]^ Copyright 2019, The Authors, published by Springer Nature.

In another study, Liu et al.^[^ ^72^ ^]^ explored the characteristics and development of ZrO_2_ films on silicon using ALD. Analysis of the atomic force microscopy (AFM) images in **Figure** **9** showed that ZrO_2_ thin films are uniform and smooth. Specifically, the root mean square (RMS) roughness value for the amorphous ZrO_2_ film deposited at 150 °C (Figure 9a) was 0.293 nm, indicating a smoother surface for amorphous films, whereas the increase in roughness (1.718 nm) upon deposition at 350 °C shown in Figure 9b suggested the formation and growth of grains in the film. After annealing at 400 and 1000 °C, the RMS roughness values for the annealed films (Figure 9c,d) remained largely unchanged, indicating sufficient crystal growth. Yet, the RMS roughness of ZrO_2_ thin films increased with the number of ALD cycles as crystal growth occurred (Figure 9e,f). This suggested that the growth was limited when the film was thin but continued grain growth occurred when the ALD cycles were increased. Further investigation in the study revealed that oxygen inside the ZrO_2_ film can diffuse toward the substrate to form an SiO_*x*_ interfacial layer during the postannealing process. This reduces the interface defect states and increases the quality of the film, effectively reducing capacitance and leakage current.

To mitigate the impact of lattice mismatch in ALD, several approaches have been proposed, including the use of buffer layers and lattice‐matching substrates.^[^ ^73^ ^]^ Interfacial layers can serve as a bridge between the substrate and the deposited film, allowing for some degree of lattice relaxation and reducing the strain energy. In addition, lattice‐matching substrates with crystal structures similar to those of the deposited material can help minimize lattice mismatch and reduce the strain energy. Ongoing research involves strain engineering, in which effect of strain due to various substrates on electronic and mechanical properties of the thin films is studied.^[^ ^74^ ^]^ Overall, minimizing the impact of lattice matching is crucial for the successful application of ALD in electronic devices. As demand for high‐quality thin films continues to increase, it is essential to develop and refine approaches that address this challenge.

### 2.5 Seeding Layers

ALD is a versatile technique for the deposition of thin films with atomic‐level precision and uniformity. The use of seed layers in ALD can significantly influence the nucleation, crystal structure, growth rate, and dielectric properties of the deposited films, making the selection of appropriate seed layers crucial. While not always necessary, seed layers can be particularly advantageous in applications in which specific properties or growth orientations are desirable. The optimization of seed layers remains an active area of research, with various strategies explored, including the use of ultrathin layers,^[^ ^75^ ^]^ in situ cleaning,^[^ ^76^ ^]^ surface modification,^[^ ^77^ ^]^ and alternative nucleation layers.^[^ ^78^ ^]^ However, the use of seed layers can also lead to the formation of interface layers, which can undermine the overall performance of a deposited film.

#### 2.5.1 Assisted Nucleation

One way to address these issues and achieve high‐quality ALD films with desirable properties is to employ nucleation layers. Fallahazad et al.^[^ ^79^ ^]^ found that oxidized titanium and aluminum films can serve as suitable nucleation layers for scaling an Al_2_O_3_ dielectric on graphene, with a significant impact on the dielectric constant (*k*) and morphology of the ALD Al_2_O_3_ in the form of k values of 5.5 and 12.7 for aluminum and titanium nucleation layers, respectively. A demonstration of G‐FETs with top dielectric stacks as thin as 2.6 nm was possible using a spatially uniform titanium nucleation layer 0.6 nm thick. Moreover, TEM analysis revealed that Al_2_O_3_ grown using a titanium interface is crystalline to some extent, whereas Al_2_O_3_ grown on aluminum is amorphous because Al nucleation layer might not be structurally compatible with the grown Al_2_O_3_ as a result of mismatched crystal structure.

**Figure 10:**
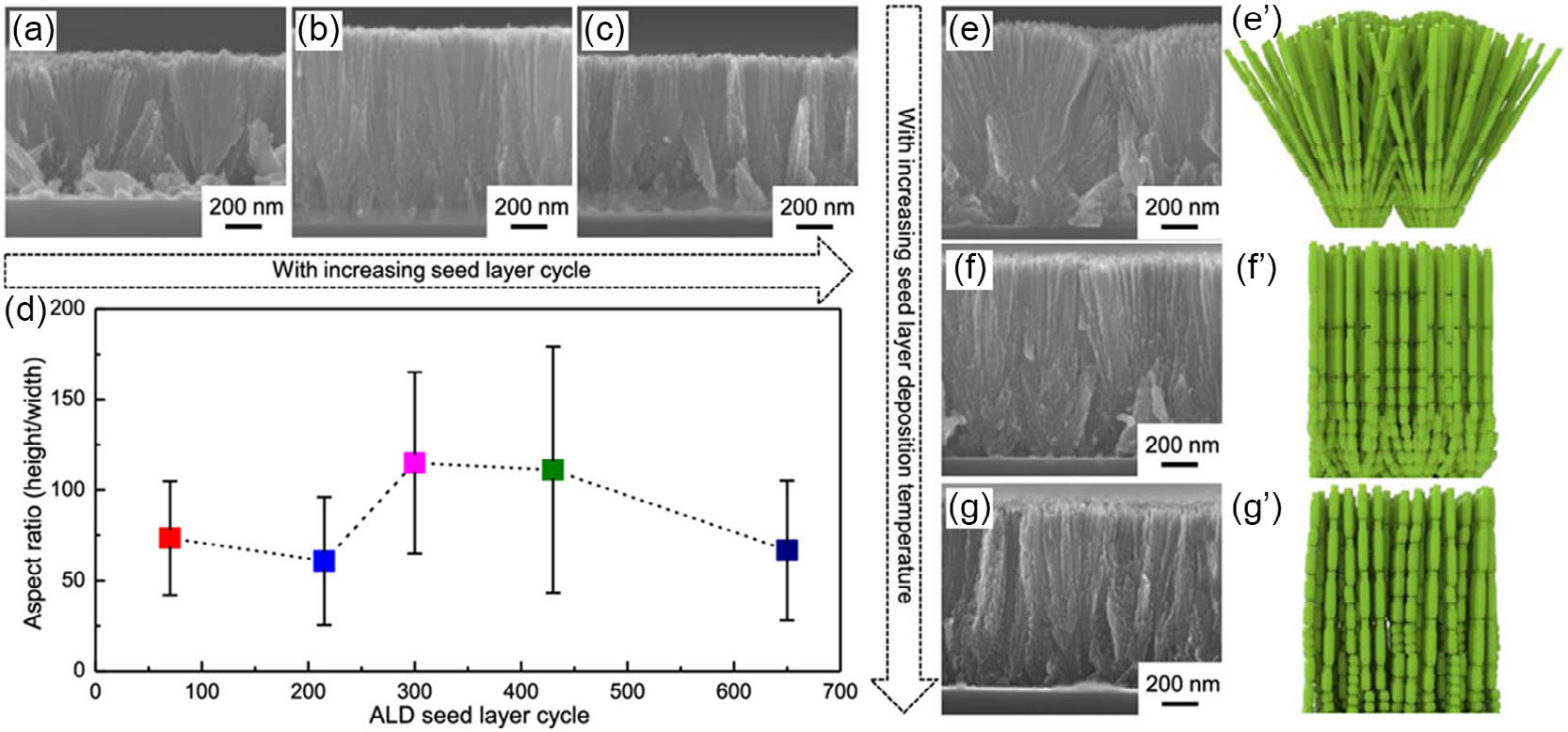
a–c) FE‐SEM images of ALD SnO_2_ nanowires with varying numbers of seed‐layer cycles, i.e., a) 215, b) 430, and c) 650, and d) their tunable aspect ratios. e,e′–g,g′) The effect of temperature is shown through FE‐SEM images and corresponding animated structure at 250 °C (e,e′), 300 °C (f,f′), and 350 °C (g,g′). a–g,g′) Reproduced with permission.^[^ ^80^ ^]^ Copyright 2020, American Chemical Society.

Apart from nucleation layers to form certain crystal structures, seed layers to generate specific morphology are also significantly researched. Bera et al.^[^ ^80^ ^]^ used ALD to seed SnO_2_ nanowires on different conducting substrates, controlling the nucleation rate and adjusting the nanowires’ characteristics. Although the nanowires were grown through solvothermal process, their morphology and properties were dependent on the underlying ALD seed layers. The study utilized several cycles (215, 430, and 650) of ALD‐SnO_2_ deposited at 300 °C as a seed layer to grow nanowires exhibiting enhanced properties via post‐ALD solvothermal method. Enhanced electrochemically active surface area and record charge‐separation efficiency were observed when nanowires were grown on carbon cloths and SnO_2_ nanosheets, respectively. The growth of SnO_2_ nanowires was examined, as shown in **Figure** **10**a–c, demonstrating the effect of SnO_2_ seed‐layer thickness. The length of the nanowires increased with seed‐layer thickness until a maximum length was achieved at 300 and 430 cycles, and then decreased at 650 cycles (Figure 10d). Furthermore, the orientation of the nanowires was influenced by the seed‐layer thickness, with thinner layers favoring (002) plane orientation and growth in small groups. As the seed‐layer thickness increased, this preference vanished. The effect of seed‐layer deposition temperature on the growth of SnO_2_ nanowires is shown in Figure 10e–g. An investigation of seed layers deposited at 250, 300, and 350 °C revealed deposition at 250 °C resulted in nanowires deviating from a perpendicular orientation, growing in groups, and forming a large branch array. As deposition temperature increased, the growth tendency decreased to medium and small branches, with the nanowires favoring (002) plane orientation, as shown in Figure 10e’–g’. These studies highlight the potential of ALD seed layers in growth of morphologies apart from conventional thin‐film depositions. Exploring orientated growth of various materials via ALD seed layers can lead toward novel and facile methods to generate new morphologies that can be used in a variety of applications such as photoanodes and photocatalysis.

#### 2.5.2 Induced Nucleation via Advanced ALD

**Figure 11:**
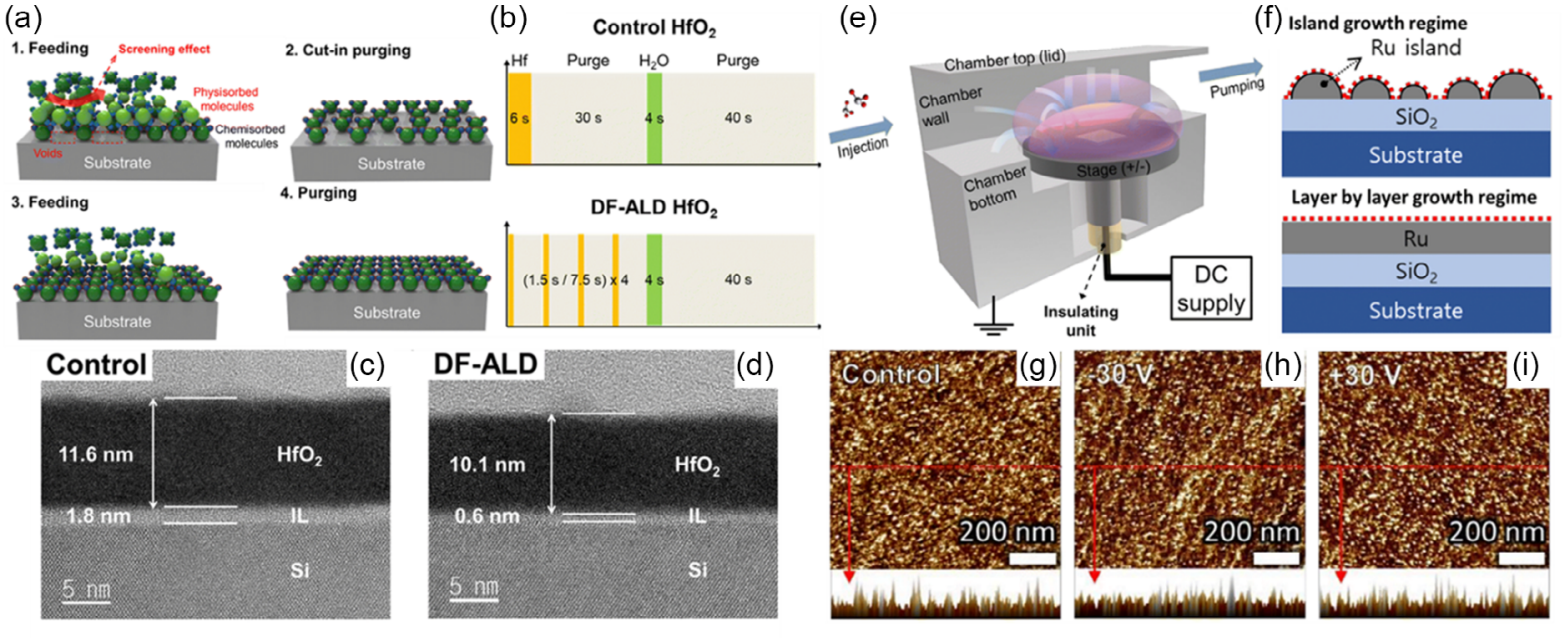
Advanced ALD processes: a) discrete‐feeding ALD showing screening effect, b) a process recipe highlighting cut‐in purge steps, and c,d) a deposited DF‐ALD HfO_2_ film compared to typical ALD. a–d) Reproduced with permission.^[^ ^82^ ^]^ Copyright 2023, Royal Society of Chemistry. e) An electric potential‐assisted ALD process chamber, f) comparison of surface area between nucleation stage and layer‐wise growth stage, and g–i) AFM images of control, positive bias, and negative bias for EA‐ALD Ru film at the nucleation stage. e–i) Reproduced with permission.^[^ ^83^ ^]^ Copyright 2023, Royal Society of Chemistry.

Recent research has focused on advanced ALD methods such as discrete feeding ALD (DF‐ALD) and electric potential‐assisted ALD (EA‐ALD), both of which allow for greater surface coverage during the initial nucleation stage of ALD. These methods essentially create seeding layers that improve film growth. This leads to higher surface coverage of reaction sites with chemisorbed precursors rather than mere physisorption. Although ideal ALD deposition assumes total coverage of the substrate surface, in practice, phenomena such as steric hindrance and screening effects come into play. Han et al.^[^ ^81^ ^]^ used a discrete feeding method (**Figure** **11**a) during ALD of ruthenium metal film to achieve high‐density nucleation sites rather than typical island‐like growth, which led to decreased grain size. Basically, a standard precursor exposure step of ALD consisting of a single pulse of a precursor is separated by several short cut‐in purge steps (Figure 11b). These purges cause removal of physisorbed precursor molecules that block potential reaction sites. The result was an ultrathin continuous ruthenium film 3 nm thick that exhibited superior properties, i.e., decreased resistivity and increased work function. Park et al.^[^ ^82^ ^]^ used the same principle of DF‐ALD‐deposited HfO_2_ films to achieve a fully saturated substrate surface, effectively reducing the interfacial layer thickness (Figure 11c,d) and the impurities in the ALD film. Han et al.^[^ ^83^ ^]^ also used electric potential to study the growth behavior of the ruthenium ALD films on a SiO_2_ substrate by supplying a DC power supply to the stage (Figure 11e). The study found that EA‐ALD affects the bonding energy of surface groups, leading to changes in ALD growth, resulting in an overall higher deposition rate. Generally, a transition point is observed during ALD between the early nucleation stage with an island‐like growth and subsequent layer‐wise growth (Figure 11f). In EA‐ALD, there is greater nucleation during the initial stage, leading to increased deposition, as confirmed by the increased number of islands revealed by AFM (i.e., 430, 450, and 500 μm^−2^ for control, positive bias, and negative bias, respectively) (Figure 11g–i). The applied electric potential could be used to alter crystal orientation, grain size, and physical density. A negative bias led to a decreased bond strength and vice versa due to changes in electron density and allowing varying degrees of precursor adsorption. A downside was higher oxygen impurities, which was expected; as the grain size decreased due to greater nucleation the grain boundaries where oxygen can easily react increased.

ALD has the potential to be utilized for versatile interfacial engineering in various practical applications. Future research in this area will need to address scalability, reproducibility, and reliability to enable the commercialization and integration of ALD into advanced device technologies.

## 3 Future Outlooks

Considering the layer‐wise growth of ALD and unavoidable use of heterogeneous multilayered materials in the modern electronics application, interactions at the interfaces are inevitable. Interfacial interactions suggest beneficial opportunities as well as challenges to resolve. The last decade has seen immense progress and innovation in this field. However, some areas require further exploration as prospects for future research:

3.1

3.1.1

##### • Exploring novel 2DEG interfaces

Discovery of 2DEG has inspired development of novel devices and implementation of options that lead to superior performance. However, there is still a need for continued exploration of other material interfaces and in‐depth study of defect levels that can induce formation of 2DEG. First‐principle studies to identify candidates can be useful and feasible.

##### • Investigating reaction chemistries for ALD of freestanding structures

In theory, the ALD processes should form an ideal film. In practice, a number of factors come into play, such as steric hindrance and screening effects. Novel techniques such as absorbate control can allow for necessary adjustments to fit actual conditions. Further insights into the mechanisms involved may be possible through in situ characterizations during ALD.

##### • Deeper understanding of surface/interface chemical reactions

From the current literature, it is evident that there is a lot to learn about what is happening at the surface and interface of the material during the ALD reactions. With postreaction characterizations, only broad assumptions can be made of the processes occurring, but computational science and in situ techniques can help us better understand these interfacial reactions on a deeper level.

##### • Screening substrates for lattice matching

Several studies indicate that the substrate plays a significant role in ALD film growth. An important but overlooked step is the choice of substrate. Screening substrates for specific ALD film growth in regard to crystal structure, orientation, film density, and more should be worthwhile subjects of future research.

##### • Widespread implementation of advanced ALD

It is clear that challenging the conventional ALD method has allowed researchers to find more practical approaches and superior outcomes. The effectiveness of DF‐ALD for the growth of ultrathin conformal films is undeniable, and its implementation in other materials should be considered. In addition, exploration of advanced ALD techniques such as DF‐ALD and EA‐ALD is an intriguing prospect.

## Conflict of Interest

The authors declare no conflict of interest.
